# Supplementary material for: Widespread stable noncanonical peptides identified by integrated analyses of ribosome profiling and ORF features
Source: Nat Commun. 2024 Mar 2;15:1932. doi: 10.1038/s41467-024-46240-9 (PMC10908861; doi:10.1038/s41467-024-46240-9)
Supplement: Supplementary file 1 — Supplementary Information [file 41467_2024_46240_MOESM1_ESM.pdf]

SUPPLEMENTARY INFORMATION

Widespread stable noncanonical peptides identified by integrated analyses of ribosome profiling and ORF features

Haiwang Yang*, Qianru Li*, Emily K. Stroup, Sheng Wang, and Zhe Ji

This PDF includes:

Supplementary Fig. 1 to 11

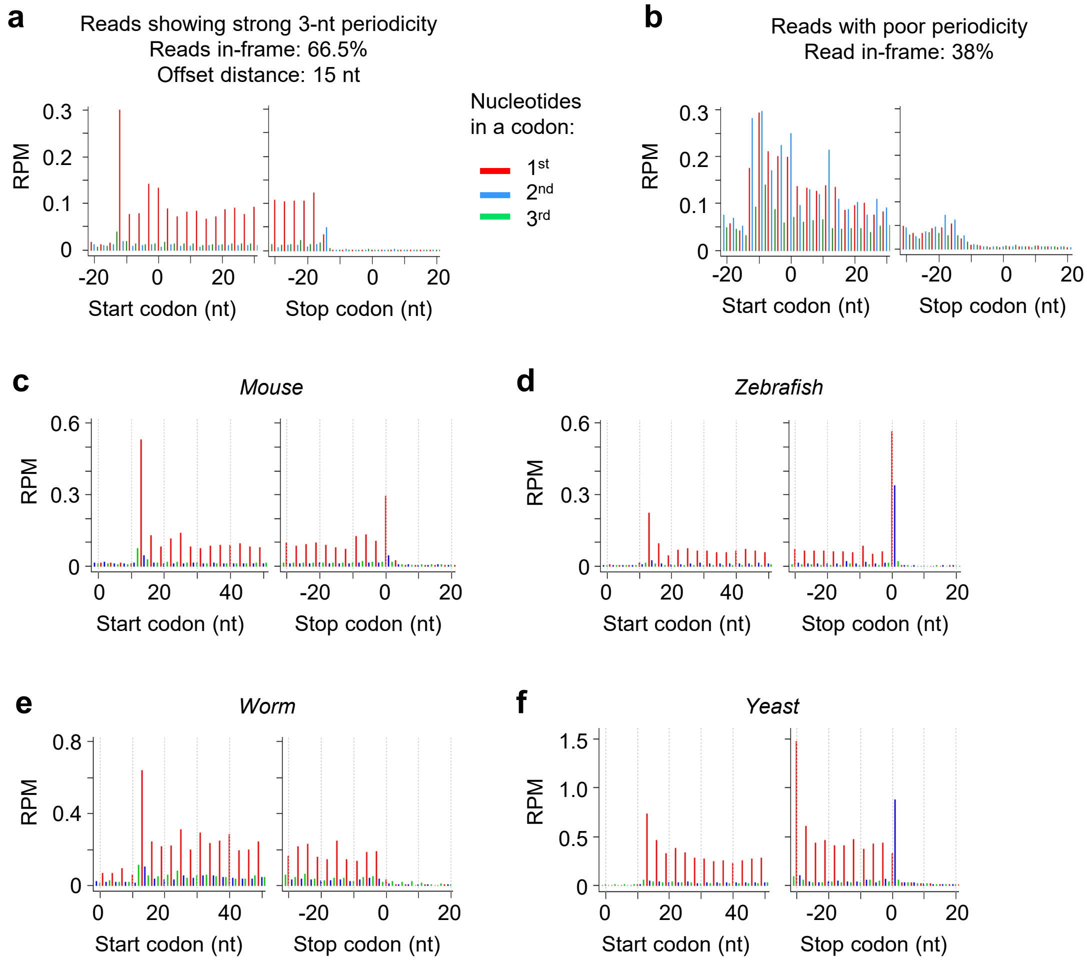

Supplementary Figure 1. The quality control of ribosome profiling datasets.
(a) Example reads showing strong 3-nt periodicity in canonical ORFs with 66.5% of reads assigned to 1st nts of codons. The read distributions around start and stop codons of canonical ORFs are shown. The y-axis represents reads per million (RPM) values. The offset distance between 5'-end of reads to ribosomal A-site is 15 nt.
(b) Example reads showing poor 3-nt periodicity.
(c-f) The ribosomal A-site adjusted high-quality ribosome profiling reads in *mouse* (c), *zebrafish* (d), *worm* (e), and *yeast* (f). These high-quality reads were included in the analyses to identify genome-wide translated ORFs.

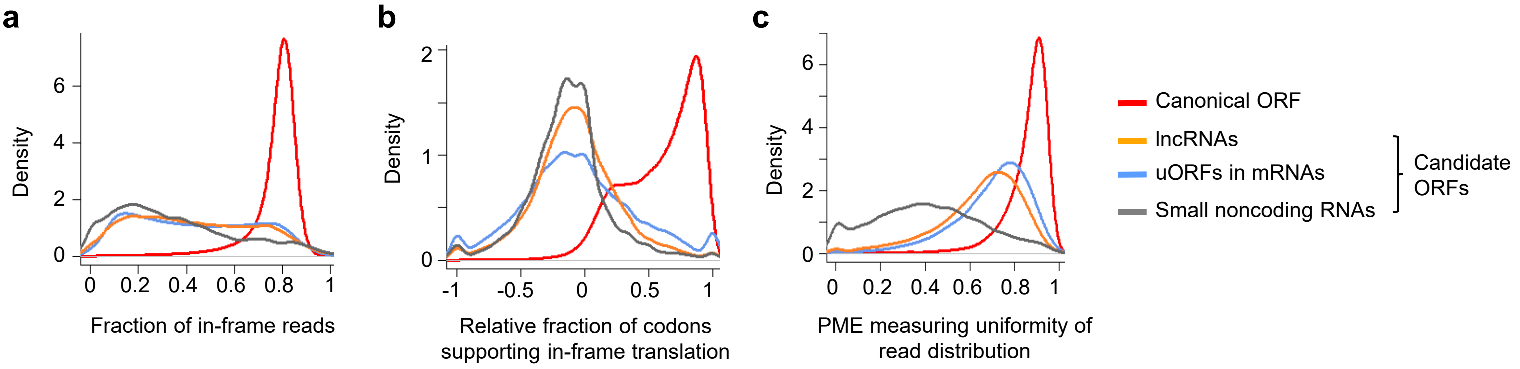

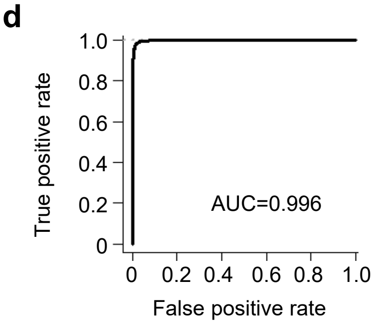

e

Feature	Coefficient	*P*-value
Fraction of in-frame reads	0.41	10^{-105}
Relative fraction of codons supporting in-frame translation	0.41	0
Uniformity of read distribution	0.47	0

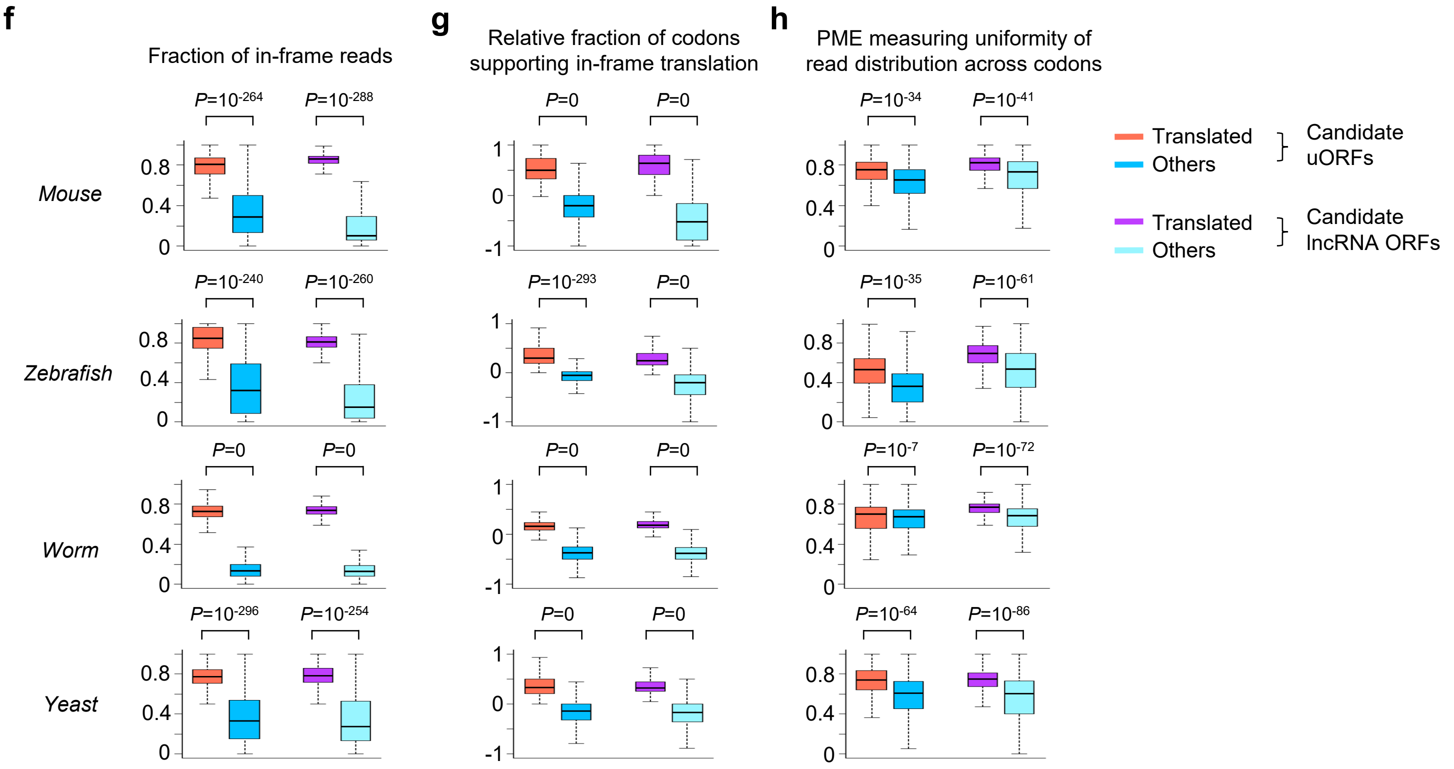

Supplementary Figure 2. RibORF identifies genome-wide translated ORFs across species.

(a) Fractions of reads in 1st nts of codons in the indicated types of ORFs.

(b) Relative fraction of codons supporting in-frame translation in the indicated types of ORFs.

(c) Distribution of PME values in the indicated types of ORFs.

(d) ROC curve to measure RibORF algorithm performance for classifying canonical ORFs vs. internal off-frame candidate ORFs in *human* ribosome profiling data.

(e) The coefficients and *P*-values of indicated features used for RibORF prediction using merged ribosome profiling reads in *humans*.

(f-h) The features used to distinguish translated ORFs vs. other candidate ORFs across *mouse*, *zebrafish*, *worm,* and *yeast*: the fraction of in-frame reads (f), the relative fraction of codons supporting in-frame translation (g), and PME measuring the uniformity of read distribution (h). The boxes are bounded by the 25 and 75 percentiles and the center represents the median. The whiskers extend from each edge of the box to indicate the 1.5x interquartile range. We randomly sampled 1,000 ORFs in each group for comparison. The two-sided Wilcoxon Rank Sum Test *P*-values comparing the translated ORFs vs. other candidate ORFs are shown.

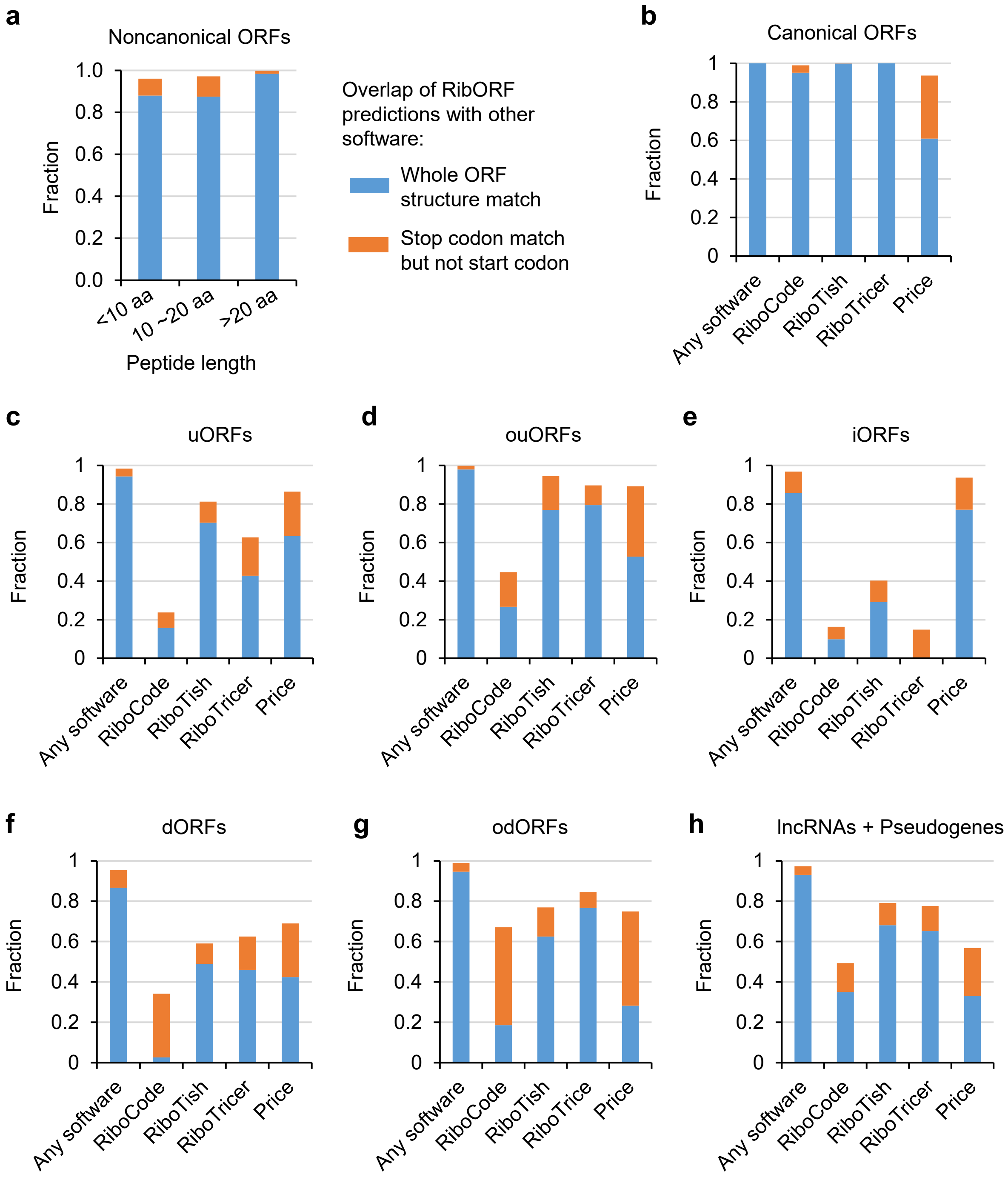

Supplementary Figure 3. Compare our RibORF predictions with other software.
(a) Fraction of RibORF-identified ncORFs also identified by other software. We grouped the ncORFs based on their lengths. We examined the fraction of ORFs showing full structure match as well as these showing stop codon match but not the start codon. (b-h) We grouped the ORFs based on their subtypes and examined the fractions of ORFs identified by other software.

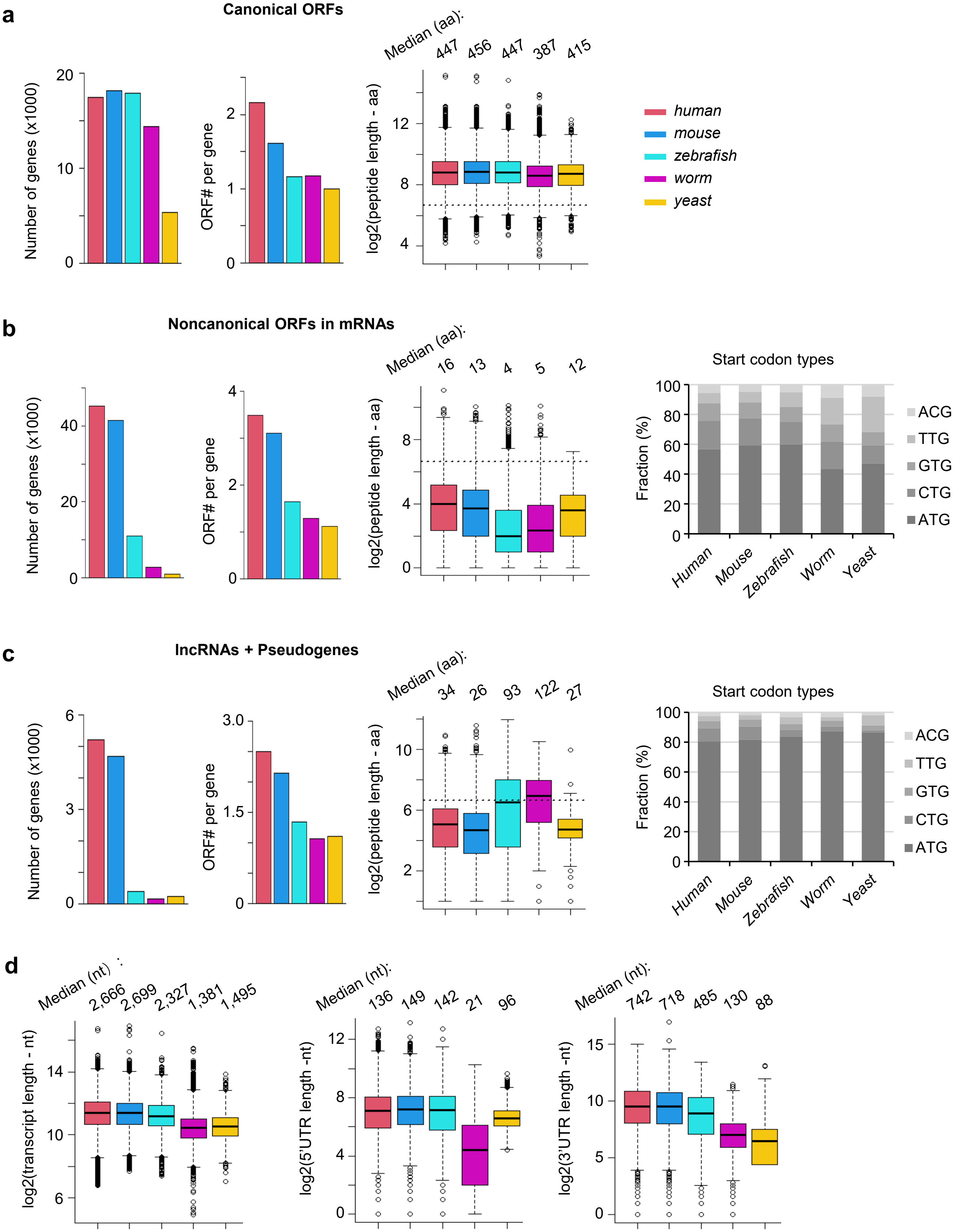

Supplementary Figure 4. Features of translated ORFs across species.
(a) The numbers of canonical ORFs and their length distribution.
(b) The numbers of ncORFs in mRNAs, their length distribution and start codon types.
(c) The numbers of ncORFs in annotated lncRNAs and pseudogenes, their length distribution and start codon types.
(d) The distribution of transcript lengths and 5'UTR/3'UTR lengths of protein-coding genes.

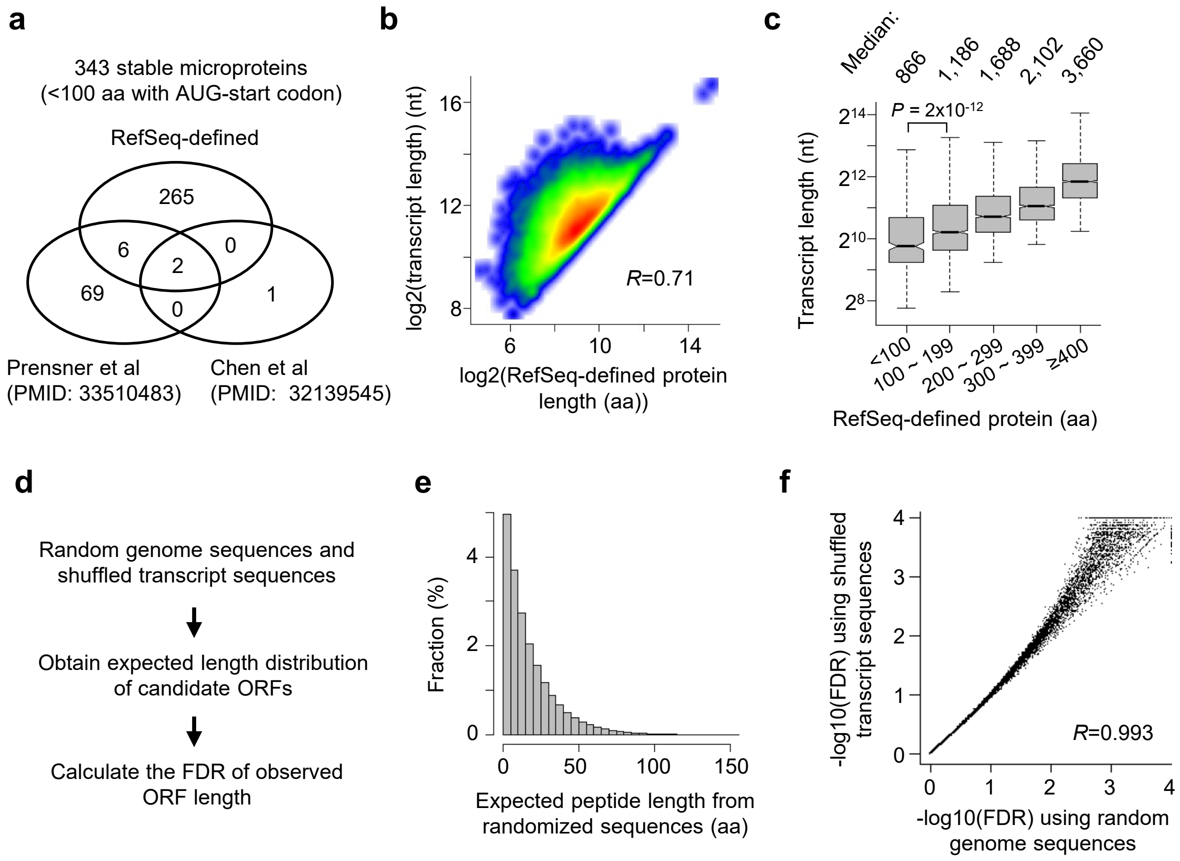

Supplementary Figure 5. The calculation of expected ORF length distribution controlling transcript lengths.
(a) The Venn diagram showing the source and overlap of stable microproteins we curated from the literature/database in this study. For RefSeq-defined microproteins, we required the longest peptide isoforms of the genes are <100 aa.
(b) The correlation between all the RefSeq-defined canonical ORF lengths and transcript lengths (N=20,695). The Pearson correlation coefficient is shown. All transcript isoforms of a gene were considered in this analyses.
(c) The distribution of transcript lengths for RefSeq-defined proteins grouped by their lengths. The two-sided Wilcox-rank sum test *P*-value comparing transcript lengths of long protein groups (100-199 aa) vs. the microproteins group (<100 aa) is shown. The boxes are bounded by the 25 and 75 percentiles and the center represents the median. The whiskers extend from each edge of the box to indicate the 1.5x interquartile range. N=377, 2155, 2815, 2969, 12379 for proteins <100 aa, 100~199 aa, 200~299 aa, 300~399 aa, and ≥400 aa, respectively.
(d) The steps for calculating the FDRs of observed ORF lengths.
(e) The length distribution of expected ORFs from randomized sequences.
(f) Comparison of the calculated length FDRs based on randomized transcripts and genome sequences. We randomly sampled 10,000 transcripts for this analysis. The Person correlation coefficient is shown.

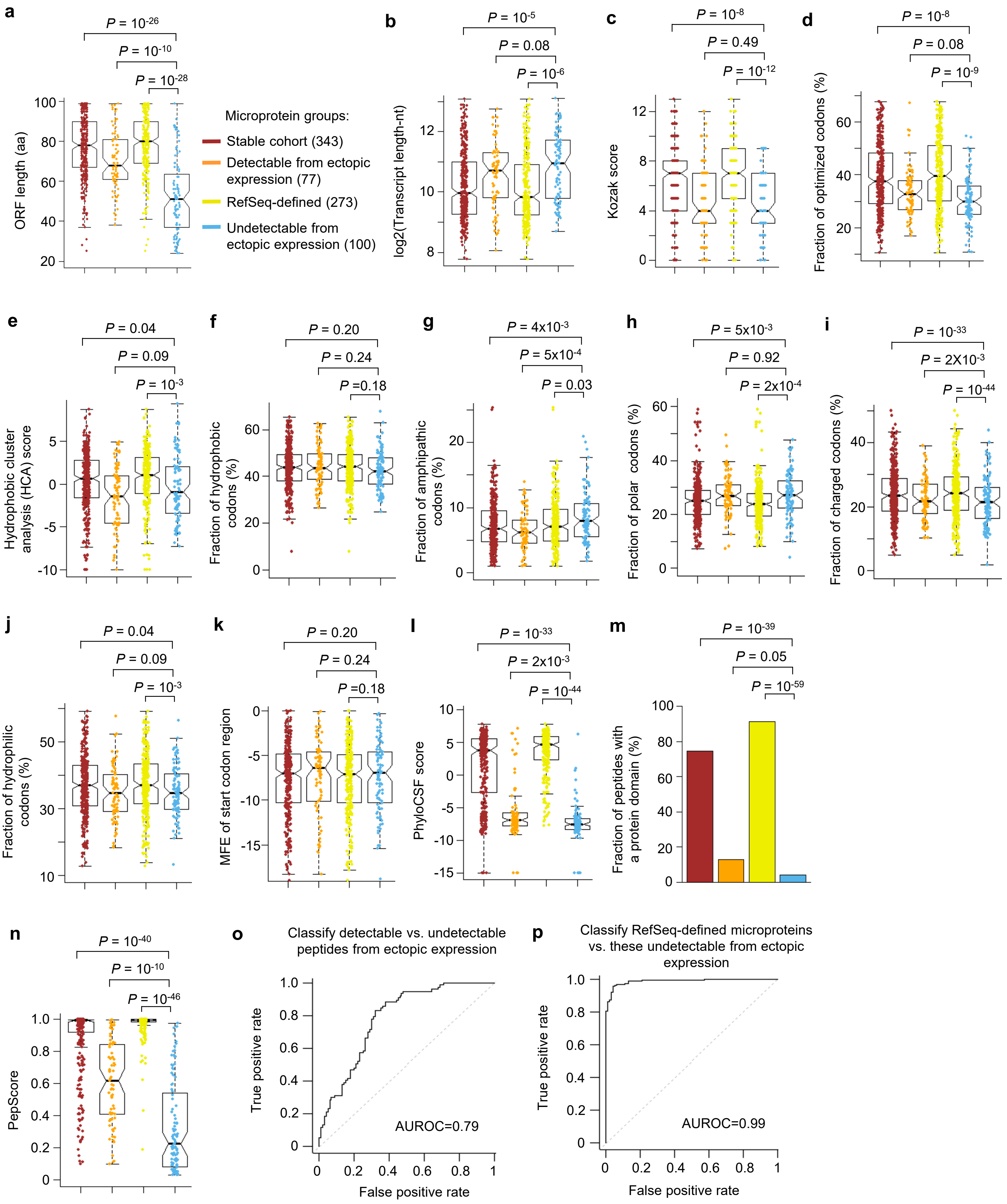
a
P = 10-26
P = 10-10
P = 10-28
ORF length (aa)
Microprotein groups:
Stable cohort (343)
Detectable from ectopic expression (77)
RefSeq-defined (273)
Undetectable from ectopic expression (100)
b
P = 10-5
P = 0.08
P = 10-6
log2(Transcript length-nt)
c
P = 10-8
P = 0.49
P = 10-12
Kozak score
d
P = 10-8
P = 0.08
P = 10-9
Fraction of optimized codons (%)
e
P = 0.04
P = 0.09
P = 10-3
Hydrophobic cluster analysis (HCA) score
f
P = 0.20
P = 0.24
P =0.18
Fraction of hydrophobic codons (%)
g
P = 4x10-3
P = 5x10-4
P = 0.03
Fraction of amphipathic codons (%)
h
P = 5x10-3
P = 0.92
P = 2x10-4
Fraction of polar codons (%)
i
P = 10-33
P = 2X10-3
P = 10-44
Fraction of charged codons (%)
j
P = 0.04
P = 0.09
P = 10-3
Fraction of hydrophilic codons (%)
k
P = 0.20
P = 0.24
P =0.18
MFE of start codon region
l
P = 10-33
P = 2x10-3
P = 10-44
PhyloCSF score
m
P = 10-39
P = 0.05
P = 10-59
Fraction of peptides with a protein domain (%)
n
P = 10-40
P = 10-10
P = 10-46
PepScore
o
Classify detectable vs. undetectable peptides from ectopic expression
True positive rate
False positive rate
AUROC=0.79
p
Classify RefSeq-defined microproteins vs. these undetectable from ectopic expression
True positive rate
False positive rate
AUROC=0.99

Supplementary Figure 6. Compare the molecular features of stable microproteins vs. those undetectable from ectopic expression. The stable microproteins were further separated into two groups, including those detectable from ectopic expression (Prensner et al, PMID: 33510483) and those annotated by the RefSeq database. The number of peptides in each group is shown in parentheses.
(a) The ORF length distribution of the peptide groups. The two-sided Wilcoxon rank-sum test *P*-values comparing different groups are shown. The boxes are bounded by the 25 and 75 percentiles and the center represents the median. The whiskers extend from each edge of the box to indicate the 1.5x interquartile range. Similarly, for panels b-l, and n.
(b) The transcript length distribution.
(c) The Kozak score of the translation initiation sites.
(d) The fraction of optimized codons.
(e) The hydrophobic cluster analysis (HCA) scores.
(f-j) The fraction of hydrophobic (f), amphipathic (g), polar (h), charged (i), and hydrophilic (j) amino acids.
(k) The minimum free energy (MFE) values measuring the structure of sequences around translation initiation sites.
(l) The distribution of PhyloCSF scores.
(m) The fraction of peptides containing a predicted domain. The two-sided Fisher's exact test *P*-values comparing different groups are shown.
(n) The distribution of PepScores.
(o) The ROC curve showing the performance using PepScore to classify the detected vs. undetected peptides from ectopic expression. The AUROC value is shown.
(p) The ROC curve showing the performance using PepScore to classify RefSeq-defined proteins vs. undetected peptides from ectopic expression. The AUROC value is shown.

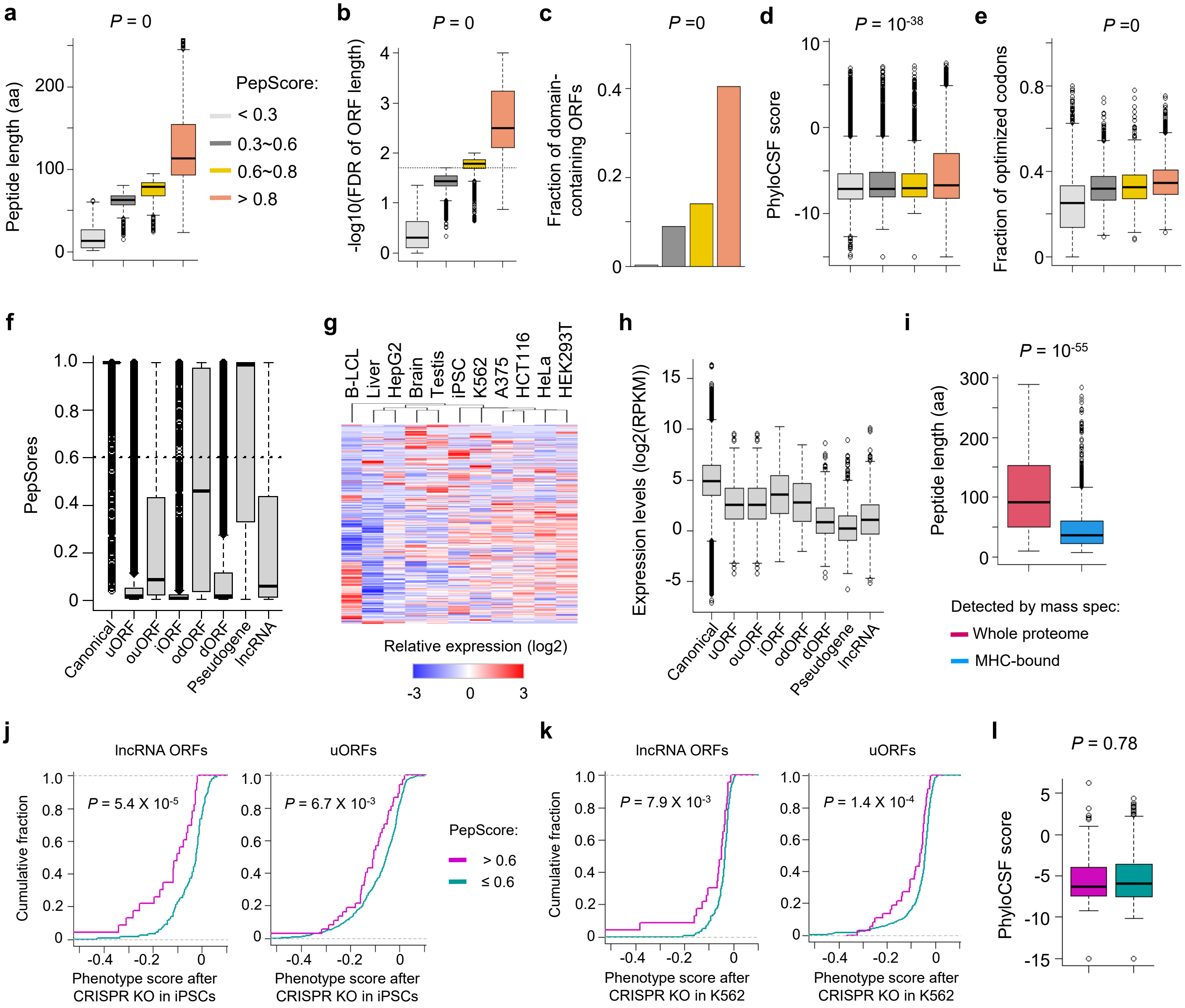

a
P = 0
Peptide length (aa)
PepScore:
< 0.3
0.3~0.6
0.6~0.8
> 0.8
b
P = 0
-log10(FDR of ORF length)
c
P =0
Fraction of domain-containing ORFs
d
P = 10-38
PhyloCSF score
e
P =0
Fraction of optimized codons
f
PepSores
Canonical
uORF
ouORF
iORF
odORF
dORF
Pseudogene
lncRNA
g
B-LCL
Liver
HepG2
Brain
Testis
iPSC
K562
A375
HCT116
HeLa
HEK293T
Relative expression (log2)
h
Expression levels (log2(RPKM))
i
P = 10-55
Peptide length (aa)
Detected by mass spec:
Whole proteome
MHC-bound
j
lncRNA ORFs
uORFs
Cumulative fraction
P = 5.4 X 10-5
P = 6.7 X 10-3
PepScore:
> 0.6
≤ 0.6
Phenotype score after CRISPR KO in iPSCs
k
lncRNA ORFs
uORFs
Cumulative fraction
P = 7.9 X 10-3
P = 1.4 X 10-4
Phenotype score after CRISPR KO in K562
l
P = 0.78
PhyloCSF score

Supplementary Figure 7. The expression and regulation of ncORFs with different PepScores.
(a-e) ncORFs were grouped based on PepScores. Then we compared the peptide lengths, the length FDRs, fractions of protein-domain containing peptides, their PhyloCSF scores, and fractions of optimized codons among the groups. The two-sided Wilcoxon rank sum test *P*-values comparing the PepScore>0.8 group vs. PepScore<0.3 are shown. N=29255, 2107, 1172, 3640 for ncORFs with PepScore <0.3, 0.3~0.6, 0.6~0.8, and >0.8, respectively. The boxes are bounded by the 25 and 75 percentiles and the center represents the median. The whiskers extend from each edge of the box to indicate the 1.5x interquartile range.
(f) Distribution of PepScores among the different types of ORFs. N=15806 for uORFs, N=2086 for ouORFs, N=3636 for iORFs, N=536 for odORFs, N=3600 for dORFs, N=1402 for pseudogene ORFs, N=9108 for lncRNA ORFs, and N=33238 for canonical ORFs. The boxplot format is the same as in (a-e).
(g) The relative expression levels of 3,011 ncORFs with high PepScores (>0.6) showing a >10-fold expression difference among the 11 indicated cell types. The data accession numbers are listed in the Methods section.
(h) The relative expression levels of ORF types with high PepScores (>0.6). The ORF numbers are shown in Figure 3. The maximum expression of an ORF across 10 cell types were used for the calculation. The boxplot format is the same as in (a-e).
(i) Comparison of the lengths of noncanonical peptides detected by mass spectrometry: whole proteome (N=326) or MHC-I bound (N=1480). The two-sided Wilcoxon rank sum test *P*-value is shown.
(j-k) The phenotype score after CRISPR knockout (KO) of lncRNA ORFs and uORFs in iPSCs and K562 cells. The ORFs were grouped based on PepScore: N=60 ncORFs with high PepScores (>0.6) and N=854 ncORFs with low PepScores (≤0.6). The two-sided Wilcoxon rank sum test *P*-values are shown.
(l) Comparison of the PhyloCSF scores of PepScore-high vs. low groups used in the above phenotype score analyses. The two-sided Wilcoxon rank sum test *P*-value is shown.

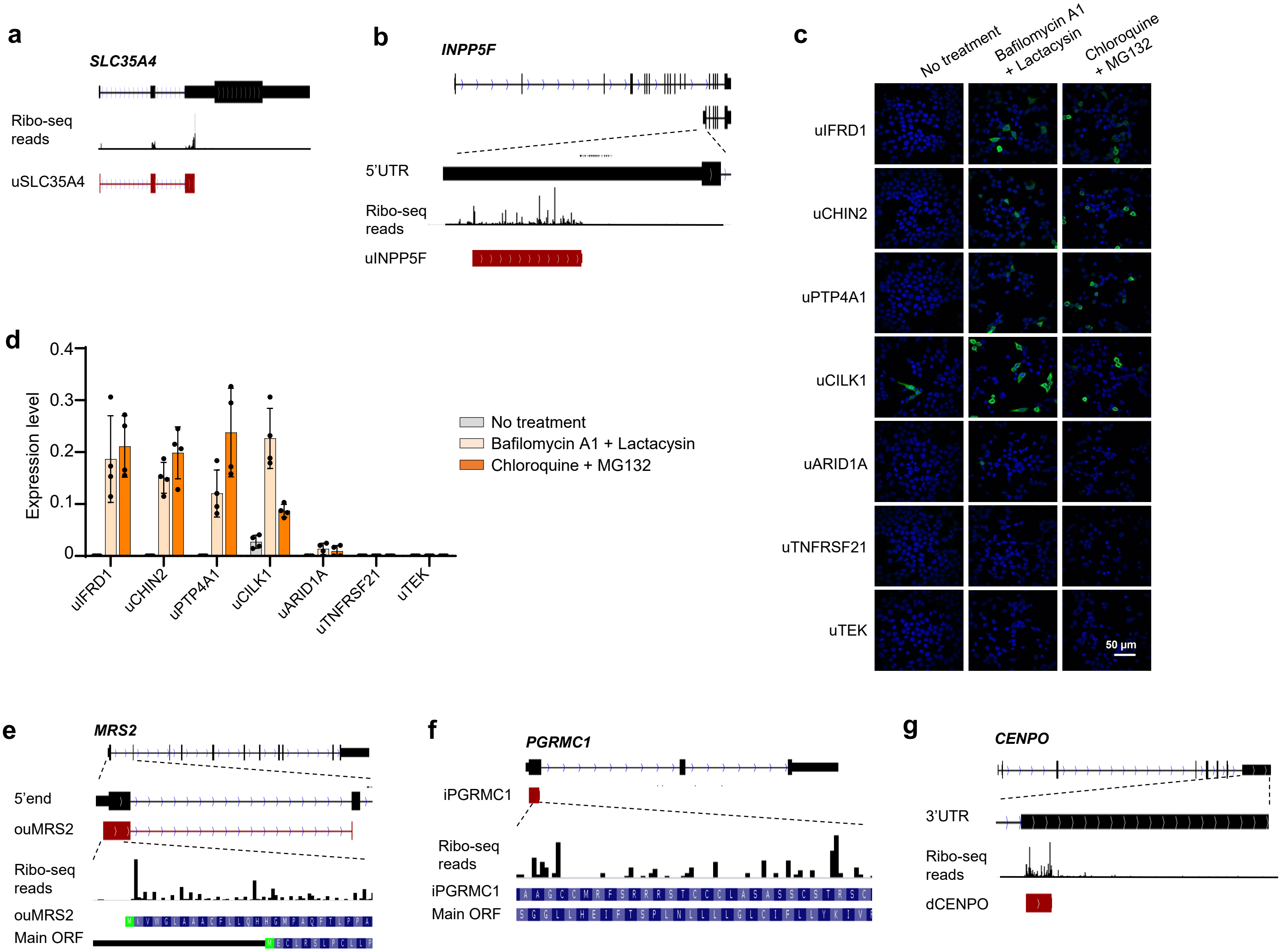

Supplementary Figure 8. Characterize the expression and stability of noncanonical peptides.
(a-b) The ribosome profiling read distribution and identified uORFs in SLC35A4 and INPP5F.
(c) The immunostaining experiments to examine the expression of uORF peptides in cells with and without treatment with the indicated proteasome and lysosome inhibitors. Expressed uORF peptides were labeled in green and cell nuclei were stained in blue. Scale bar, 50 μm.
(d) The summarized expression of uORF peptides shown in (c). Data are shown as mean values ± SD of four replicates and are representative of three independent experiments. Peptide expression level can be found in Supplementary Table S9.
(e-g) The ribosome profiling read distribution and identified translated ORFs in MRS2, PGRMC1 and CENPO.

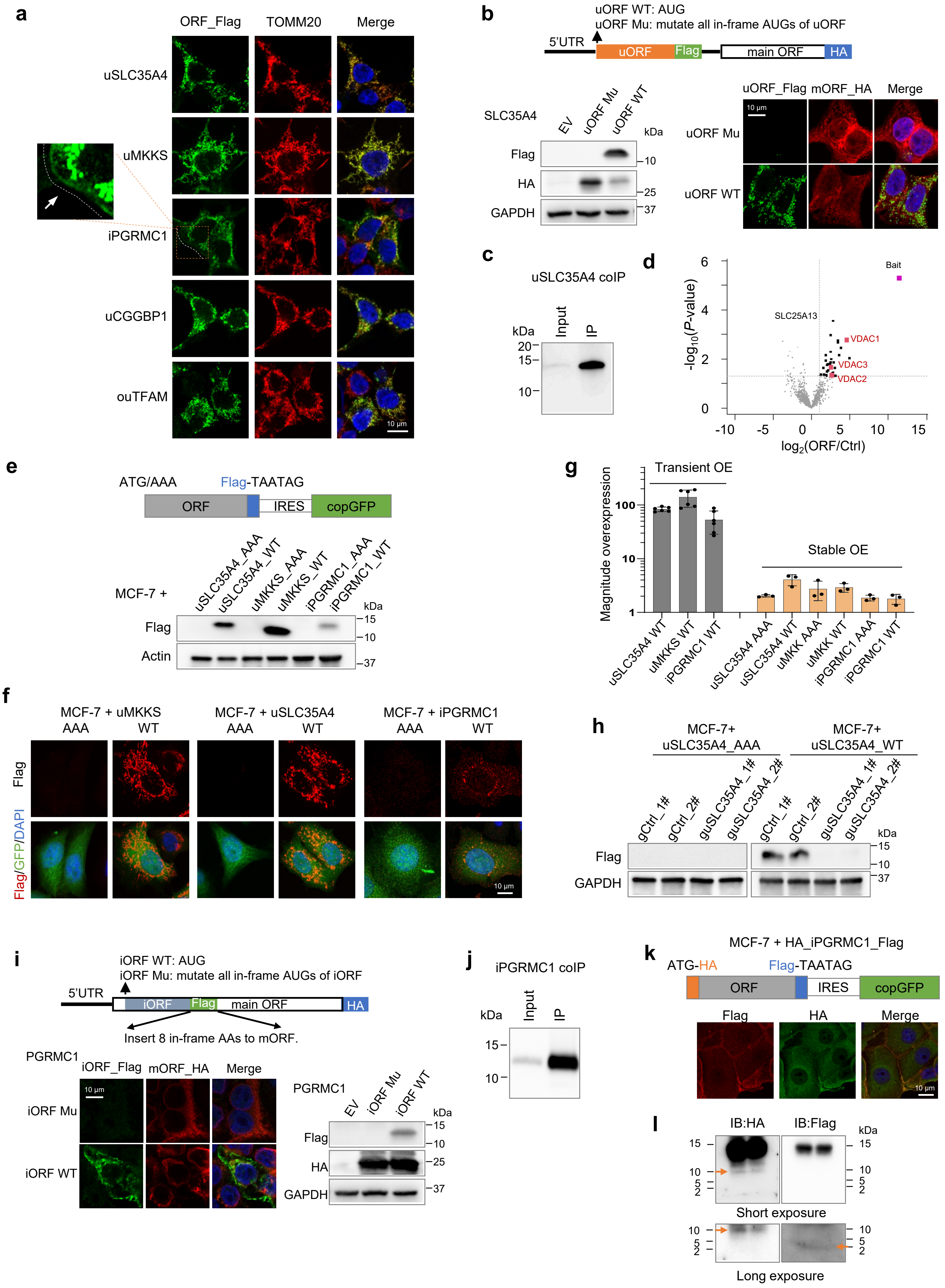

a
ORF_Flag
TOMM20
Merge
uSLC35A4
uMKKS
iPGRMC1
uCGGBP1
ouTFAM
10 μm
b
uORF WT: AUG
uORF Mu: mutate all in-frame AUGs of uORF
5'UTR
uORF
Flag
main ORF
HA
SLC35A4
EV
uORF Mu
uORF WT
kDa
Flag
HA
GAPDH
10
25
37
uORF_Flag
mORF_HA
Merge
uORF Mu
uORF WT
10 μm
c
uSLC35A4 coIP
Input
IP
kDa
20
15
10
d
Bait
SLC25A13
VDAC1
VDAC3
VDAC2
-log10(P-value)
log2(ORF/Ctrl)
e
ATG/AAA
Flag-TAATAG
ORF
IRES
copGFP
MCF-7 +
uSLC35A4_AAA
uSLC35A4_WT
uMKKS_AAA
uMKKS_WT
iPGRMC1_AAA
iPGRMC1_WT
Flag
Actin
g
Transient OE
Stable OE
Magnitude overexpression
uSLC35A4 WT
uMKKS WT
iPGRMC1 WT
uSLC35A4 AAA
uSLC35A4 WT
uMKK AAA
uMKK WT
iPGRMC1 AAA
iPGRMC1 WT
f
MCF-7 + uMKKS
MCF-7 + uSLC35A4
MCF-7 + iPGRMC1
AAA
WT
Flag
Flag/GFP/DAPI
h
MCF-7+ uSLC35A4_AAA
MCF-7+ uSLC35A4_WT
gCtrl_1#
gCtrl_2#
guSLC35A4_1#
guSLC35A4_2#
Flag
GAPDH
i
iORF WT: AUG
iORF Mu: mutate all in-frame AUGs of iORF
5'UTR
iORF
Flag
main ORF
HA
Insert 8 in-frame AAs to mORF.
PGRMC1
iORF_Flag
mORF_HA
Merge
iORF Mu
iORF WT
EV
iORF Mu
iORF WT
Flag
HA
GAPDH
j
iPGRMC1 coIP
Input
IP
k
MCF-7 + HA_iPGRMC1_Flag
ATG-HA
Flag-TAATAG
ORF
IRES
copGFP
Flag
HA
Merge
l
IB:HA
IB:Flag
Short exposure
Long exposure

Supplementary Figure 9. Examine the expression and biological roles of mitochondria-localized noncanonical peptides.
(a) Peptides encoded by uSLC35A5, uMKKS, iPGRMC1, uCGGBP1, and ouTFAM are localized to mitochondria. Ectopically expressed Flag-tagged ORFs were co-immunostained with TOMM20. iPGRMC1 peptide also shows cell membrane localization, indicated by an orange arrow. Scale bar, 10 μm.
(b) Expression of Flag-tagged uSLC35A4 and HA-tagged SLC35A4 (main ORF) in the native transcript. uSLC35A4 (green) and SLC35A4 (red) show different subcellular localizations. Scale bar, 10 μm.
(c-d) Co-IP mass spectrometry of uSLC35A5 peptide. The enrichment of uSLC35A5 was validated in (c). Co-IP proteins are shown in(d). The bait is labeled in magenta (d). Top interacting proteins are labeled with gene names (two-sided T-test, n = 3 independent experiments).
(e-f) Stable expression of uSLC35A5, uMKKS, and iPGRMC1 in MCF-7 cell lines was validated by western blotting (e), and their mitochondrial localization was validated using immunostaining (f). The cells expressing start codon-mutated ORF sequences (ORF_AAA) were used as negative controls. Scale bar, 10 μm.
(g) The magnitude of overexpression for the ectopically expressed constructs comparing to endogenous transcript levels, as quantified by qPCR. Data are shown as mean values ± SD of six replicates of transient overexpression and three replicates of stable overexpression.
(h) Knockout of uSLC35A5 in MCF-7 stable cell lines.
(i) Expression of HA-tagged PGRMC1 (main ORF) and Flag-tagged iPGRMC1 in the native transcript. The flag tag induced an insertion of 8 in-frame amino acids into the main ORF without changing the protein sequences. iPGRMC1 (green) was expressed and localized to a distinct cellular compartment compared to PGRMC1 (red). Scale bar, 10 μm.
(j) Immunoblotting showing the enrichment of iPGRMC1 peptide in the co-IP lysate.
(k) Stable expression of N-terminal and C-terminal dual tagged Ipgrmc1 in MCF-7. Pseudocolored immunostaining images show that C-terminal Flag-tagged iPGRMC1 peptide is enriched on the cell membrane, while N-terminal HA-tagged iPGRMC1 peptides accumulate in the cytosol.
(l) Cleaved iPGRMC1 peptides detected by immunoblotting. The cleavage products are indicated using the brown arrows.
Experiments (a-i) were performed at least three times with similar results. Source data are provided as a Source Data file.

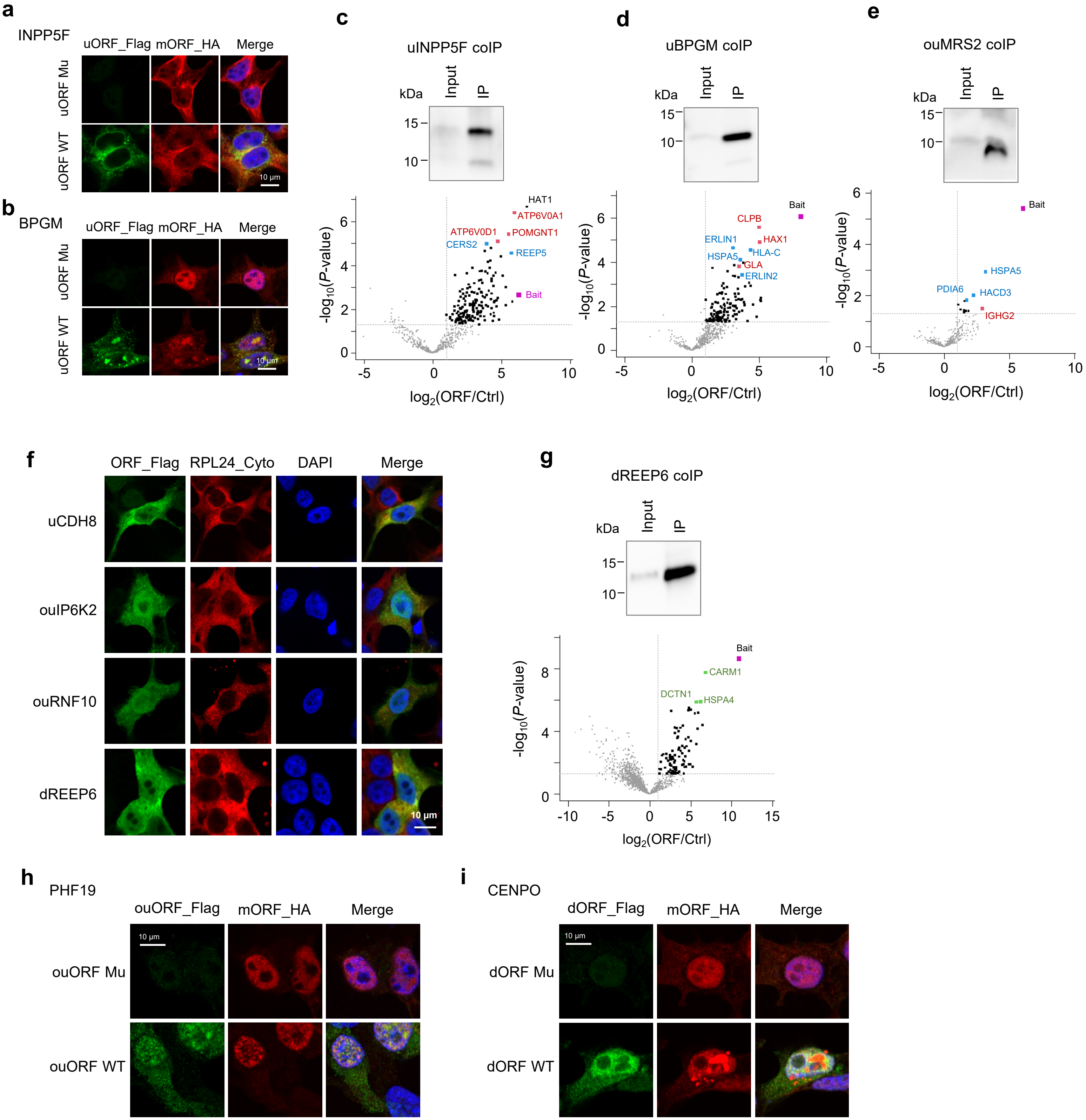
a
INPP5F
uORF_Flag
mORF_HA
Merge
uORF Mu
uORF WT
10 μm
b
BPGM
uORF_Flag
mORF_HA
Merge
uORF Mu
uORF WT
10 μm
c
uINPP5F coIP
kDa
Input
IP
15
10
HAT1
ATP6V0A1
ATP6V0D1
POMGNT1
CERS2
REEP5
Bait
-log10(P-value)
log2(ORF/Ctrl)
d
uBPGM coIP
kDa
Input
IP
15
10
Bait
CLPB
ERLIN1
HAX1
HSPA5
HLA-C
GLA
ERLIN2
-log10(P-value)
log2(ORF/Ctrl)
e
ouMRS2 coIP
kDa
Input
IP
15
10
Bait
HSPA5
PDIA6
HACD3
IGHG2
-log10(P-value)
log2(ORF/Ctrl)
f
ORF_Flag
RPL24_Cyto
DAPI
Merge
uCDH8
ouIP6K2
ouRNF10
dREEP6
10 μm
g
dREEP6 coIP
kDa
Input
IP
15
10
Bait
CARM1
DCTN1
HSPA4
-log10(P-value)
log2(ORF/Ctrl)
h
PHF19
ouORF_Flag
mORF_HA
Merge
ouORF Mu
ouORF WT
10 μm
i
CENPO
dORF_Flag
mORF_HA
Merge
dORF Mu
dORF WT
10 μm

Supplementary Figure 10. Examine the expression of noncanonical peptides localized to ER and cytosol.
(a-b) Immunostaining showing the expression of uINPP5F, uBPGM and main ORFs in the native transcript context (Fig. 6c). Flag-tagged uORF and HA-tagged main ORF were ectopically expressed in HEK293T cells. uORF peptide (green), main ORF (red) and DAPI (blue) were co-immunostained. Scale bar, 10 μm.
(c-e) Co-IP mass spectrometry experiments identifying uINPP5F-, uBPGM-, and ouMRS2- interaction proteins. The enrichment of ORF peptides was validated with western blotting. The co-IP proteins are shown in the volcano plots. The bait is labeled in magenta. Top enriched ER proteins (blue) and secretory proteins (red) are colored and marked with gene names (two-sided T-test, n = 3 independent experiments).
(f) Flag-tagged ORFs were ectopically expressed in HEK293T cells and co-immunostained for Flag (green), cytosolic marker RPL24 (red), and DAPI (blue). Scale bar, 10 μm.
(g) Co-IP mass spectrometry results for the dREEP6 peptide. The enrichment of dREEP6 peptide in the lysate was validated with western blotting. Co-IP proteins are shown in the volcano plot. The bait is labeled in magenta. Top interacting proteins showing cytosolic/nuclear distribution are labeled (green) with gene names (two-sided T-test, n = 3 independent experiments).
(h-i) Immunostaining showing the expression of ouPHF19, dCENPO and main ORFs in the native transcript. Flag-tagged ncORF and HA-tagged main ORF were ectopically expressed in HEK293T cells. ouORF/dORF (green), main ORFs (red) and DAPI (blue) were co-immunostained. Scale bar, 10 μm. Source data are provided as a Source Data file.

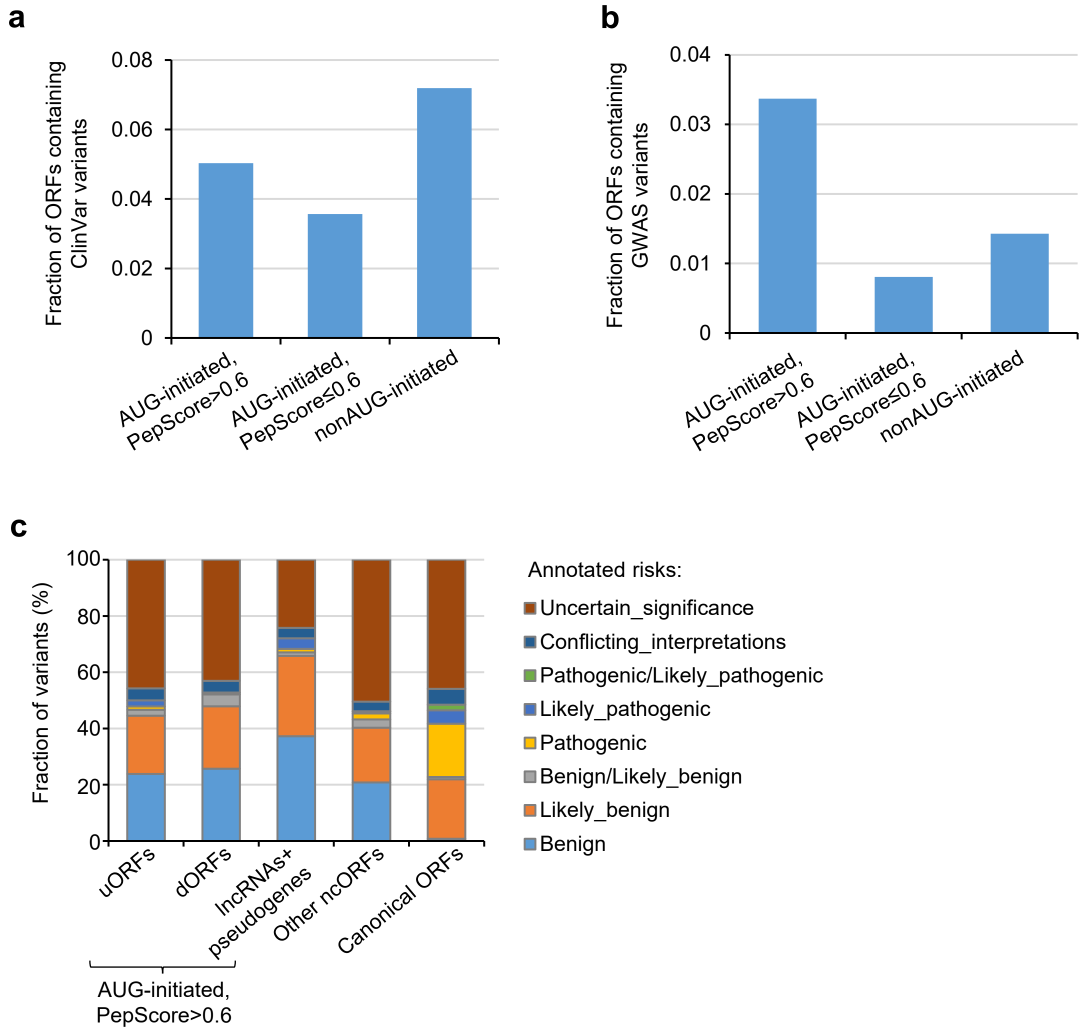

Supplementary Figure 11. Analyses of ClinVar and GWAS variants in ncORFs.

(a) Fraction of ncORFs containing ClinVar variants. ncORFs were grouped based on PepScores and start codon types.

(b) Fraction of ncORFs containing GWAS variants.

(c) The distribution of annotated risks of ClinVar variants located in different ORF regions.
