# Supplementary material for: Widespread stable noncanonical peptides identified by integrated analyses of ribosome profiling and ORF features
Source: Nat Commun. 2024 Mar 2;15:1932. doi: 10.1038/s41467-024-46240-9 (PMC10908861; doi:10.1038/s41467-024-46240-9)
Supplement: Supplementary file 3 — Inventory of Supplementary Information [file 41467_2024_46240_MOESM3_ESM.docx]

**SUPPORTING INFORMATION**

Supplementary Fig. 1. The quality control of ribosome profiling datasets.

Supplementary Fig. 2. RibORF identifies genome-wide translated ORFs across species.

Supplementary Fig. 3. Compare our RibORF predictions with other software.

Supplementary Fig. 4. Features of translated ORFs across species.

Supplementary Fig. 5. The calculation of expected ORF length distribution controlling transcript lengths.

Supplementary Fig. 6. Compare the molecular features of stable microproteins vs. those undetectable from ectopic expression.

Supplementary Fig. 7. The expression and regulation of ncORFs with different PepScores.

Supplementary Fig. 8. Characterize the expression and stability of noncanonical peptides.

Supplementary Fig. 9. Examine the expression and biological roles of mitochondria-localized noncanonical peptides.

Supplementary Fig. 10. Examine the expression of noncanonical peptides localized to ER and cytosol.

Supplementary Fig. 11. Analyses of ClinVar and GWAS variants in ncORFs.

Supplementary Data 1. The ribosome profiling datasets analyzed in this study. We show the GSE accession numbers of analyzed datasets. For reads showing strong 3-nt periodicity, we show the sample accession number, sample description, fragment size, read number, the fraction of in-frame reads, and adjusted offset distance between read 5’-end to ribosomal A-site.

Supplementary Data 2. The RibORF model parameters and associated AUROC values used to identify translated ORFs using different ribosome profiling datasets.

Supplementary Data 3. RibORF-identified genome-wide translated ORFs across different species. The ORF structure is presented in the Bed format. The genomic coordinates were based on the following genome assembly: *human* (hg38) and *mouse* (mm10), *zebrafish* (GRCz11), *worm* (WBcel235), and *yeast* (sacCer3).

Supplementary Data 4. The expression levels of translated ORFs across different samples. For each sample name, we annotated the cell type and associated database accession number. The Salmon software was used to calculate the isoform specific ORF expression levels.

Supplementary Data 5. The comparison between our RibORF predictions and other software. For each ORF, we annotated whether it could be identified by another software with the following annotation. 2: full ORF structure match; 1: stop codon match but start codon mismatch; 0: no match.

Supplementary Data 6. The collection of characterized microproteins used to build the PepScore model in this study. The stable and undetectable microproteins were shown in two different spreadsheets.

Supplementary Data 7. The PepScores for AUG-initiated *human* noncanonical peptides.

Supplementary Data 8. The ORF sequences, primers, CRISPR gRNAs, and antibodies used in this study.

Supplementary Data 9. The expression levels of ORF peptides from ectopic expression experiments.

Supplementary Data 10. The ncRNAs with ClinVar and GWAS variants shown in the VCF format. To accommodate the VCF, the ORF IDs were shown as gene symbol:transcript ID:start:end:ORF type. For the GWAS variants, we showed the trait types and associated -log10(*P*-value). For the ClinVar variants, their annotated risks are also shown.
