# Supplementary material for: Widespread stable noncanonical peptides identified by integrated analyses of ribosome profiling and ORF features
Source: Nat Commun. 2024 Mar 2;15:1932. doi: 10.1038/s41467-024-46240-9 (PMC10908861; doi:10.1038/s41467-024-46240-9)
Supplement: Supplementary file 14 — Reporting Summary [file 41467_2024_46240_MOESM14_ESM.pdf]

nature portfolio

Corresponding author(s): Zhe Ji

Last updated by author(s): Feb 1, 2024

Reporting Summary

Nature Portfolio wishes to improve the reproducibility of the work that we publish. This form provides structure for consistency and transparency in reporting. For further information on Nature Portfolio policies, see our Editorial Policies and the Editorial Policy Checklist.

Statistics

For all statistical analyses, confirm that the following items are present in the figure legend, table legend, main text, or Methods section.

n/a	Confirmed	
☐	☒	The exact sample size (*n*) for each experimental group/condition, given as a discrete number and unit of measurement
☐	☒	A statement on whether measurements were taken from distinct samples or whether the same sample was measured repeatedly
☐	☒	The statistical test(s) used AND whether they are one- or two-sided<br>*Only common tests should be described solely by name; describe more complex techniques in the Methods section.*
☐	☒	A description of all covariates tested
☐	☒	A description of any assumptions or corrections, such as tests of normality and adjustment for multiple comparisons
☐	☒	A full description of the statistical parameters including central tendency (e.g. means) or other basic estimates (e.g. regression coefficient) AND variation (e.g. standard deviation) or associated estimates of uncertainty (e.g. confidence intervals)
☐	☒	For null hypothesis testing, the test statistic (e.g. *F*, *t*, *r*) with confidence intervals, effect sizes, degrees of freedom and *P* value noted<br>*Give P values as exact values whenever suitable.*
☒	☐	For Bayesian analysis, information on the choice of priors and Markov chain Monte Carlo settings
☒	☐	For hierarchical and complex designs, identification of the appropriate level for tests and full reporting of outcomes
☐	☒	Estimates of effect sizes (e.g. Cohen's *d*, Pearson's *r*), indicating how they were calculated

Our web collection on statistics for biologists contains articles on many of the points above.

Software and code

Policy information about availability of computer code

Data collection	Not applicable.
Data analysis	Bowtie2 (v2.2.6), TopHat (v2.1.0), HTSeq (v0.9.1), samtools (v1.9), deeptools (v3.1.1), salmon (v1.6.0), R (v3.5.1), ViennaRNA (v2.5.0), pyHCA, tmhmm (v2.0c), deeploc (v1.0), pfam_scan (v1.6-2), snpEff (v4.5), MaxQuant (v1.6.17.0), Perseus (v1.6.15.0), STAR (v2.1.0), DAVID (2021), GraphPad Prism (v9.2.0), ImageJ (v1.52), blastp (v2.6.0). The codes developed in this study is available at: https://github.com/zhejilab/RibORF and https://github.com/zhejilab/PepScore.

For manuscripts utilizing custom algorithms or software that are central to the research but not yet described in published literature, software must be made available to editors and reviewers. We strongly encourage code deposition in a community repository (e.g. GitHub). See the Nature Portfolio guidelines for submitting code & software for further information.

Data

Policy information about availability of data

All manuscripts must include a data availability statement. This statement should provide the following information, where applicable:

- Accession codes, unique identifiers, or web links for publicly available datasets
- A description of any restrictions on data availability
- For clinical datasets or third party data, please ensure that the statement adheres to our policy

We analyzed a large-cohort of published ribosome profiling datasets and their accession numbers are listed in Table S1. The sequencing datasets generated in this

study are available in the Gene Expression Omnibus (GEO) repository with the accession number GSE216093. The mass spectrometry data were deposited in Proteomics Identifications Database (PRIDE) with the project accession PXD037658.

Research involving human participants, their data, or biological material

Policy information about studies with human participants or human data. See also policy information about sex, gender (identity/presentation), and sexual orientation and race, ethnicity and racism.

Reporting on sex and gender	Not applicable.
Reporting on race, ethnicity, or other socially relevant groupings	Not applicable.
Population characteristics	Not applicable.
Recruitment	Not applicable.
Ethics oversight	Not applicable.

Note that full information on the approval of the study protocol must also be provided in the manuscript.

Field-specific reporting

Please select the one below that is the best fit for your research. If you are not sure, read the appropriate sections before making your selection.

☒ Life sciences ☐ Behavioural & social sciences ☐ Ecological, evolutionary & environmental sciences

For a reference copy of the document with all sections, see nature.com/documents/nr-reporting-summary-flat.pdf

Life sciences study design

All studies must disclose on these points even when the disclosure is negative.

Sample size	We analyzed datasets from the public available databases and generated our own data as well. Sample size for each analyses is indicated in figure legends and methods.
Data exclusions	No data were excluded from the analyses.
Replication	For bench experiments, we replicated our biological findings for at least 3 times. For RNA-seq and mass spectrometry experiments, at least 2 biological replicates were performed. For computational analyses, random sampling approaches were used for some analyses. For each analysis, we presented the sample size, mean, and p-value when applicable. And we ensured that the findings were reproducible.
Randomization	For some analyses, we randomly sampled a subset of ORFs for the model training and testing, respectively. The procedures and analyses steps were described in the method section. When random sampling was used, we set the random seed for reproducibility.
Blinding	To build our models, the testing datasets for algorithm performances were independent of the data used for model training. 5-fold cross-validation was used for the model training. For bench experiments, the control and treatment samples were processed side by side to remove batch effects.

Reporting for specific materials, systems and methods

We require information from authors about some types of materials, experimental systems and methods used in many studies. Here, indicate whether each material, system or method listed is relevant to your study. If you are not sure if a list item applies to your research, read the appropriate section before selecting a response.

Materials & experimental systems

n/a	Involved in the study	
☐	☒	Antibodies
☐	☒	Eukaryotic cell lines
☒	☐	Palaeontology and archaeology
☒	☐	Animals and other organisms
☒	☐	Clinical data
☒	☐	Dual use research of concern
☒	☐	Plants

Methods

n/a	Involved in the study	
☒	☐	ChIP-seq
☒	☐	Flow cytometry
☒	☐	MRI-based neuroimaging

Antibodies

Antibodies used

Flag, Sigma, catalog No:F1804
HA, Abcam, catalog No:ab9110
TOMM20, Abcam, catalog No:ab186735
AIF, Cell Signaling Technology, catalog No:5318T
OXCT1, Proteintech, catalog No:12175-1-AP
VDAC1/3, Abcam, catalog No:ab14734
GAPDH, Santa Cruz, catalog No:sc-47724
PMPCA, Proteintech, catalog No:26536-1-AP
PMPCB, Proteintech, catalog No:16064-1-AP
Calnexin, Cell Signaling Technology, catalog No:2679S
Bip, Cell Signaling Technology, catalog No:3177T
MT-ND1, Abcam, catalog No:ab222892
RPS24, Proteintech, catalog No:14831-1-AP
RPL24, Proteintech, catalog No:17082-1-AP
CD63, Invitrogrn, catalog No:10628D
anti-rabbit IgG, Cell Signaling Technology, catalog No:7074S
anti-mouse IgG, Cell Signaling Technology, catalog No:7070S
Alexa Fluor 488 Goat anti-Mouse, Invitrogrn, catalog No:A32723
Alexa Fluor 555 Goat anti-Rabbit, Invitrogrn, catalog No:A21428
Alexa Fluor 555 Goat anti-Mouse, Invitrogrn, catalog No:A21424
Alexa Fluor 647 Goat anti-Rabbit, Invitrogrn, catalog No:A32733

Validation

Western blotting
Flag
https://www.sigmaaldrich.com/US/en/product/sigma/f1804?utm_source=google&utm_medium=cpc&utm_campaign=8906396310&utm_content=91369514764&gclid=Cj0KCQiAn-2tBhDVARIsAGmStVnIc2sotC60C1tiSDqoIp4YwYtDXjdsn0EHBW_hIZfaDlecxBwh2IAaAnPBEALw_wcB#product-documentation
HA
https://www.abcam.com/products/primary-antibodies/ha-tag-antibody-chip-grade-ab9110.html
TOMM20
https://www.abcam.com/products/primary-antibodies/tomm20-antibody-epr15581-54-mitochondrial-marker-ab186735.html
OXCT1
https://www.ptglab.com/products/SCOT-Antibody-12175-1-AP.htm
VDAC1/3
https://www.abcam.com/products/primary-antibodies/vdac1porin--vdac3-antibody-20b12af2-ab14734.html
GAPDH
https://www.scbt.com/p/gapdh-antibody-0411
PMPCA
https://www.ptglab.com/products/PMPCA-Antibody-26536-1-AP.htm
PMPCB
https://www.ptglab.com/products/PMPCB-Antibody-16064-1-AP.htm
Calnexin
https://www.cellsignal.com/products/primary-antibodies/calnexin-c5c9-rabbit-mab/2679
Bip
https://www.cellsignal.com/products/primary-antibodies/bip-c50b12-rabbit-mab/3177
MT-ND1
https://www.abcam.com/products/primary-antibodies/mt-nd1-antibody-ab222892.html
RPS24
https://www.ptglab.com/products/RPS24-Antibody-14831-1-AP.htm
CD63
https://www.thermofisher.com/antibody/product/CD63-Antibody-clone-Ts63-Monoclonal/10628D?gclid=Cj0KCQiAn-2tBhDVARIsAGmStVn2v6FapvFdAWed4aJNzhX9-8aCKlfL5g9ILQ-K_rDsBbP6krGHCDUaAr0xEALw_wcB&ef_id=Cj0KCQiAn-2tBhDVARIsAGmStVn2v6FapvFdAWed4aJNzhX9-8aCKlfL5g9ILQ-K_rDsBbP6krGHCDUaAr0xEALw_wcB:G:s&s_kwcid=AL!3652!3!668848227489!!!g!!!16893189572!157390419772&cid=bid_sap_cep_r01_co_cp0000_pjt0000_bid00000_0se_gaw_dy_awa_con&gad_source=1
β-Actin
https://www.cellsignal.com/products/primary-antibodies/b-actin-8h10d10-mouse-mab/3700
anti-rabbit IgG
https://www.cellsignal.com/products/secondary-antibodies/anti-rabbit-igg-hrp-linked-antibody/7074
anti-mouse IgG
https://www.cellsignal.com/products/secondary-antibodies/anti-mouse-igg-hrp-linked-antibody/7076

Immunofluorescence staining
Alexa Fluor 488 Goat anti-Mouse
https://www.thermofisher.com/antibody/product/Goat-anti-Mouse-IgG-H-L-Highly-Cross-Adsorbed-Secondary-Antibody-Polyclonal/A32723
Alexa Fluor 555 Goat anti-Rabbit
https://www.thermofisher.com/antibody/product/Goat-anti-Rabbit-IgG-H-L-Cross-Adsorbed-Secondary-Antibody-Polyclonal/A-21428
Alexa Fluor 555 Goat anti-Mouse
https://www.thermofisher.com/antibody/product/Goat-anti-Mouse-IgG-H-L-Highly-Cross-Adsorbed-Secondary-Antibody-Polyclonal/A-21424

Alexa Fluor 647 Goat anti-Rabbit
https://www.thermofisher.com/antibody/product/Goat-anti-Rabbit-IgG-H-L-Highly-Cross-Adsorbed-Secondary-Antibody-Polyclonal/A32733
Flag
https://www.sigmaaldrich.com/US/en/product/sigma/f1804?utm_source=google&utm_medium=cpc&utm_campaign=8906396310&utm_content=91369514764&gclid=Cj0KCQiAn-2tBhDVARIsAGmStVnIc2sotC60C1tiSDqoIp4YwYtDXjdsn0EHBW_hIZfaDlecxBwh2IAaAnPBEALw_wcB#product-documentation
HA
https://www.abcam.com/products/primary-antibodies/ha-tag-antibody-chip-grade-ab9110.html
TOMM20
https://www.abcam.com/products/primary-antibodies/tomm20-antibody-epr15581-54-mitochondrial-marker-ab186735.html
AIF
https://www.cellsignal.com/products/primary-antibodies/aif-d39d2-xp-rabbit-mab/5318
OXCT1
https://www.ptglab.com/products/SCOT-Antibody-12175-1-AP.htm
Calnexin
https://www.cellsignal.com/products/primary-antibodies/calnexin-c5c9-rabbit-mab/2679
RPL24
https://www.ptglab.com/products/RPL24-Antibody-17082-1-AP.htm

Eukaryotic cell lines

Policy information about cell lines and Sex and Gender in Research

Cell line source(s)	HEK293T and MCF-7 cells are purchased from ATCC (American Type Culture Collection).
Authentication	Cell lines were not authenticated.
Mycoplasma contamination	All cell lines used in this study were tested negative of mycoplasma contamination.
Commonly misidentified lines (See ICLAC register)	No commonly misidentified line was used.

Plants

Seed stocks	Not applicable.
Novel plant genotypes	Not applicable.
Authentication	Not applicable.
